# Stromal Antigen 2 Deficiency Induces Interferon Responses and Restricts Porcine Deltacoronavirus Infection

**DOI:** 10.3390/v14081783

**Published:** 2022-08-15

**Authors:** Yang Wu, Hongling Zhang, Jianfei Chen, Zhaorong Shi, Mingwei Li, Ying Zhao, Hongyan Shi, Da Shi, Longjun Guo, Li Feng

**Affiliations:** State Key Laboratory of Veterinary Biotechnology, Harbin Veterinary Research Institute, Chinese Academy of Agricultural Sciences, Harbin 150001, China

**Keywords:** PDCoV, STAG2, replication, interferon signaling pathway

## Abstract

Porcine deltacoronavirus (PDCoV) is a recently discovered enteropathogenic coronavirus and has caused significant economic impacts on the pork industry. Although studies have partly uncovered the molecular mechanism of PDCoV–host interaction, it requires further research. In this study, we explored the roles of Stromal Antigen 2 (STAG2) in PDCoV infection. We found that STAG2-deficient cells inhibited infection with vesicular stomatitis virus (VSV) and PDCoV, whereas restoration of STAG2 expression in STAG2-depleted (STAG2^−^^/^^−^) IPEC-J2 cells line restored PDCoV infection, suggesting that STAG2 is involved in the PDCoV replication. Furthermore, we found that STAG2 deficiency results in robust interferon (IFN) expression. Subsequently, we found that STAG2 deficiency results in the activation of JAK-STAT signaling and the expression of IFN stimulated gene (ISG), which establish an antiviral state. Taken together, the depletion of STAG2 activates the JAK-STAT signaling and induces the expression of ISG, thereby inhibiting PDCoV replication. Our study provides new insights and potential therapeutic targets for unraveling the mechanism of PDCoV replication.

## 1. Introduction

Porcine deltacoronavirus (PDCoV) is a recently discovered enteropathogenic coronavirus and has caused significant economic impacts on the pork industry [1,2,3]. PDCoV, similar to other swine enteric coronaviruses, including transmissible gastroenteritis virus (TGEV) and porcine epidemic diarrhea virus (PEDV), have caused frequent occurrences of diarrhea, vomiting, and dehydration in piglets [2,4,5,6,7,8]. Clinically, PDCoV infection commonly occurs in the form of co-infection with PEDV or TGEV, which has caused significant economic losses to the global swine industry. PDCoV have the potential for cross-species transmission and are causing huge economic losses in the pig industry in China and the world, which therefore needs to be urgently addressed [9].

Innate immunity plays a crucial role in host defense against invading pathogens [10,11]. During viral infection, the innate immune response is often activated, leading to the induction of the type I interferon (IFN-I or IFN α/β). IFN-I is the potent cytokine of critical importance in controlling viral infections and priming adaptive immune responses [12,13]. Following production, IFN-I initiates a positive feed-back loop by binding to their cognate receptors on the cell surface in an autocrine and paracrine manner [14,15] and activates JAK protein tyrosine kinases (JAK1 and Tyk2) which phosphorylate signal transducers and activators of transcription (STAT) 1 (STAT1) and (STAT) 2 (STAT2). STAT1 and STAT2 together with interferon regulatory factor 9 (IRF9) form a transcription factor complex termed IFN-stimulated gene factor 3 (ISGF3). Then, ISGF3 is translocated into the nucleus and binds to the IFN-stimulated response elements (ISRE) to induce the expression of IFN-stimulated genes (ISGs), which establish an antiviral state [15,16,17,18].

Cohesin is a highly-conserved protein complex that plays important roles in sister chromatid cohesion, chromatin structure, gene expression, and DNA repair [19]. In humans, cohesin is a ubiquitously expressed, multi-subunit protein complex composed of core subunits SMC1A, SMC3, RAD21, STAG1/2 and regulatory subunits WAPL, PDS5A/B, CDCA5, NIPBL, and MAU2 [20]. Recent studies have demonstrated that genes encoding cohesin subunits are somatically mutated in a wide range of human cancers [21,22]. Stromal Antigen 2 (STAG2) is the most commonly mutated subunit, and in a recent analysis was identified as one of only 12 genes that are significantly mutated in four or more cancer types. Numerous studies have demonstrated that STAG2 mutation is a common and important event in the pathogenesis of diverse human cancers [21,23,24]. Studies have demonstrated that cohesion STAG2 also has the function of transcriptional coactivation, which can enhance NF-κB-driven transcription. Meanwhile, the activity of the tumor necrosis factor alpha, the CD69, and the human immunodeficiency virus long terminal repeat promoters were enhanced by STAG2. And analysis was identified that recruitment of other components of the transcriptional co-activation complexes also depends on the interaction between STAG2 and the NF-κB subunit p65 [25]. Above all, it is apparent that the effects of STAG2 on transcriptional activation and the occurrence of some cancer types.

A novel role of STAG2 as a crucial component of the innate immune response was reported, suggesting STAG2 deficiency induces interferon responses via cGAS-STING pathway and restricts virus infection. Cohesion deficiency can cause host genomic DNA damage and increase the levels of cytoplasmic DNA, then which enter the cGAS-STING DNA-sensing pathway to stimulate IFN production and induce the activation of JAK-STAT signaling pathway. Ultimately, these processes induce the expression of ISG, they also make the host cells enter a state of antiviral and render cells resistant to rotavirus and other RNA virus infection [26]. Based on these related studies, whether STAG2 also has an effect on coronavirus replication. In the present study, we confirmed that the loss of STAG2, an important component of the cohesin complex, confers resistance to vesicular stomatitis virus (VSV) and PDCoV replication in cell culture. Mechanistically, STAG2 deficiency results in robust IFN expression and ISG expression via the activation of JAK-STAT signaling, thereby inhibiting PDCoV replication. 

## 2. Materials and Methods

### 2.1. Cell Culture and Viruses

HEK293T cells (human embryonic kidney epithelial cells; ATCC) (CRL-3216) and IPEC-J2 cells (porcine small intestine epithelial cell clone J2, donated by Yanming Zhang of Northwest A&F University, Yangling, China) [27] were cultured in Dulbecco’s minimum essential medium (DMEM) (Life Technologies, Carlsbad, CA, USA) supplemented with 10% heat-inactivated fetal bovine serum (FBS) (Gibco, Grand Island, NY, USA), 100 U/ml penicillin, 100 μg/ml streptomycin in an incubator with 5% CO_2_ at 37 °C (Thermo Scientific, Waltham, MA, USA). PDCoV strain NH (GenBanK: KU981062.1) was prepared and titrated as previously described [28]. 

### 2.2. Plasmids and Antibodies

The full-length sequence of STAG2 was constructed into the pCAGGS-HA vector to obtain the recombinant plasmid of pCAGGS-HA-STAG2. The PCR primers were designed by Primer 5. SgRNAs were designed according to the website http://crispr.mit.edu (accessed on 7 January 2020). The primer sequences and sgRNAs are listed in Table 1. All plasmid construct was confirmed by sequencing.

The listed antibodies were used in this study including the STAG2 rabbit monoclonal antibody (mAb) (5882) and a phospho-STAT1 (Tyr701) (D4A7) rabbit mAb (7649) were purchased from Cell Signaling Technology, IRDye-conjugated secondary antibody (926-32213 and 926-68072) was purchased from Li-Cor Biosciences and β-actin mouse mAb (A5441) was purchased from Sigma (St. Louis, MO, USA).

### 2.3. Virus Infection

Monolayers of IPEC-J2 cells were infected with PDCoV at an MOI of 1 for 1 h at 37 °C. Unbound virus was removed, and cells were maintained in complete medium for various time points until samples were harvested. 

### 2.4. Transfection

Cells were transfected with indicated plasmids using X-tremeGENE transfection reagent according to manufacturer’s instruction (Roche, Indianapolis, IN, USA). At the indicated times, cell samples were collected and lysed in RIPA buffer (Beyotime, Nantong, China) for Western blot analysis of target proteins. 

### 2.5. CRISPR-Cas9 Knockout Cells

STAG2-Cas9 knockout cells were generated by using the CRISPR/Cas9 system. Briefly, the designed single guide RNA (sgRNA) targeting the porcine STAG2 gene was cloned into the Lentiviral vector2 vector. pMD2.G and psPAX2, producing the VSV-G glycoprotein and envelope proteins of the lentivirus, respectively, combined with lenti-guide-puro-sgRNA-STAG2 were co-transfected into HEK293T cells to produce the recombined lentivirus. IPEC-J2 cells were infected with lentivirus, and puromycin (2.5 µg/mL) was added to select the positive clones. The monoclonal cells were obtained with the limited dilution method. Finally, the knockout of STAG2 was confirmed by Western blot at the protein level.

### 2.6. IFA

IFA was performed as described previously with slight modification [17]. Briefly, STAG2-depleted IPEC-J2 cells lines or WT cells were infected with PDCoV for 24 h, and the cells were fixed and stained with anti-PDCoV-N mouse monoclonal antibody [29] for one hour. After the removal of unbound antibodies, the cells were stained with FITC-conjugated goat anti-mouse IgG for another hour, followed by nuclei staining with DAPI (4,6-diamidino-2-phenylindole; Sigma). After washing the cells, the fluorescence was visualized with an Olympus inverted fluorescence microscope equipped with a camera.

### 2.7. Western Blot

Western blot analysis was performed as previously described [30]. Treated samples were lysed in RIPA buffer containing protease inhibitor cocktail and phosphatase inhibitors (Roche) and separated by SDS-PAGE under reducing conditions and transferred onto a PVDF membrane (Merck Millipore, Temecula, CA, USA). After blocking, the membranes were incubated with a primary antibody and then probed with an appropriate IRDye-conjugated secondary antibody (LiCor Bio-Sciences, Lincoln, NE, USA). The membranes were scanned using an Odyssey instrument (Li-Cor Biosciences) according to the manufacturer’s instructions. 

### 2.8. Quantitative RT-PCR

Quantitative RT-PCR analysis was carried out as described previously [31]. Total RNA was extracted from cells and subjected to quantitative RT-PCR using specific primers as listed in Table 1. Relative gene quantification was performed by the method of 2(-Delta Delta C(T)) [32]. 

### 2.9. TCID_50_ Assay

Collected virus samples were frozen and thawed three times and clarified by centrifugation at 8000× *g* for 10 min prior to titration. TCID_50_ assays were performed according to the method of Reed & Muench as previously described [32]. Briefly, cell monolayers were inoculated with 10-fold serial dilutions of each virus stock and incubated for 4 days prior to observation of the presence of cytopathic effect.

### 2.10. Cell Viability Assay

Cell viabilities were assessed using a cell counting kit-8 (CCK-8) (Cat NO. GK10001, GLPBIO, Montclair, CA, USA). Assays were performed according to the manufacturer’s instructions. Briefly, the WT cells and the STAG2^−^^/^^−^ cells were incubated in 96-well plates and the cell viabilities were measured at 12 h, 24 h, and 36 h. A total of 10 µL of CCK-8 reagents were added to each well of the plates, and the cells were incubated at 37 °C for 1 h, then the absorbance at 450 nm was measured by a microplate reader.

### 2.11. Statistical Analysis

Variables are expressed as mean ± S.D. Statistical analyzes were performed using student’s *t*-test. Significance is denoted in the figures as follows: *, *p* < 0.05 and **, *p* < 0.01.

## 3. Results

### 3.1. Establishment of STAG2-Knockout IPEC-J2 Cell Line

**Figure 1:**
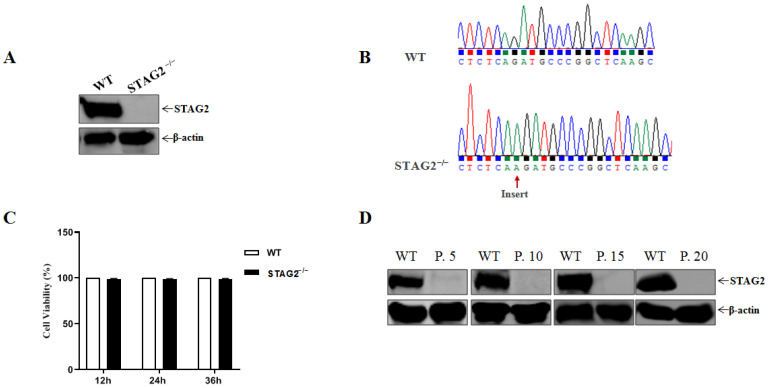
Establishment of STAG2-depleted IPEC-J2 cells line. (**A**) Western blot analysis for STAG2 expression in IPEC-J2 cell line; (**B**) Gene sequence alignment revealed that were heterozygous for STAG2 knockout alleles generated using CRISPR-Cas9 gene editing; (**C**) Cell viability was determined using CCK-8 detection; (**D**) Western blot analysis for STAG2 expression of WT IPEC-J2, STAG2^−^^/^^−^ IPEC-J2 passage 5 (P. 5), passage 10 (P. 10), passage 15 (P. 15) and passage 20 (P. 20).

To study the role of STAG2 in PDCoV infection, we then generated a single clonal STAG2 knockout in IPEC-J2 cells, porcine intestinal epithelial cell (IEC) line commonly used for PDCoV studies. The knockout effect of STAG2 was determined by Western blot, and the results revealed that the STAG2 protein was knocked out (Figure 1A). Sanger sequencing confirmed the presence of 1 bp insert in STAG2-depleted (STAG2^−^^/^^−^) IPEC-J2 cells line (Figure 1B). Meanwhile, knockout of STAG2 had no significant effect on the cell viability when compared to the wild type (WT) cells (Figure 1C). To study the function of STAG2 in PDCoV infection, we generated a stable STAG2^−^^/^^−^ IPEC-J2 cells line, STAG2^−^^/^^−^ IPEC-J2 cells were propagated and the deletion was confirmed by Western blot up to the last passage (Figure 1D).

### 3.2. Confirmation of STAG2 as a Critical Host Factor for VSV Infection

**Figure 2:**
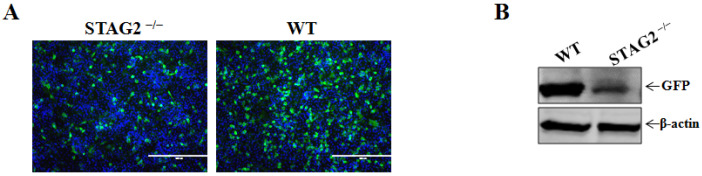
Confirmation of STAG2 as a critical host factor for VSV infection. (**A**) WT and STAG2^−^^/^^−^ were infected with VSV (MOI = 0.1). The VSV-GFP was visualized at 10 h post infection with an Olympus inverted fluorescence microscope equipped with a camera; (**B**) WT and STAG2^−^^/^^−^ were infected with VSV (MOI = 0.1). Cell samples were harvested and subjected to immunoblotting with antibodies as indicated.

We next used GFP-expressing VSV to infect WT or STAG2^−^^/^^−^ IPEC-J2 cells. Fluorescence microscopy images showed that VSV infection was evident in WT cells, and conversely VSV infection was significantly inhibited in STAG2^−^^/^^−^ cells compared to WT cells (Figure 2A). Additionally, the loss of STAG2 resulted in decreased VSV replication, as detected by comparing the level of GFP protein in VSV-infected STAG2^−^^/^^−^ cells to that in the WT cells (Figure 2B), and the results suggested that VSV was reduced in the absence of STAG2.

### 3.3. Confirmation of STAG2 as a Critical Host Factor for PDCoV Infection

**Figure 3:**
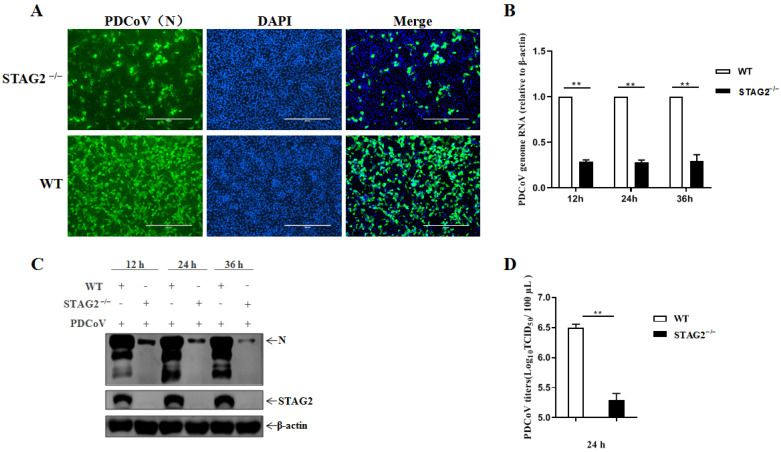
STAG2 is involved in the replication of PDCoV. (**A**) WT and STAG2^−^^/^^−^ cells were infected with PDCoV at an MOI of 1, twenty four hours post infection, cells were fixed and stained with anti-PDCoV-N mouse monoclonal antibody followed by probe with FITC-conjugated goat anti-mouse IgG. The representative results were displayed by three different channels (PDCoV-N, DAPI, and overlay) for each treatment; (**B**) WT and STAG2^−^^/^^−^ cells were infected with PDCoV at an MOI of 1, PDCoV N mRNA concentration was determined by quantitative RT-PCR at indicated time points post infection; (**C**) WT and STAG2^−^^/^^−^ cells were infected with PDCoV at an MOI of 1 followed by samples detection by Western blot at indicated time points post infection; (**D**) WT and STAG2^−^^/^^−^ cells were infected with PDCoV at an MOI of 1, twenty four hours post infection, virus yield was measured by TCID_50_. The results are representative of three independent experiments (the means ± SD). **, *p* < 0.01. The *p* value was calculated using Student’s *t*-tests.

To assess how STAG2 responds to PDCoV infection, We next used PDCoV to infect STAG2^−^^/^^−^ or WT cells. PDCoV infection was significantly inhibited in the STAG2^−^^/^^−^ cells compared with the infection in the WT cells, according to the IFA, demonstrating that PDCoV was reduced in the absence of STAG2 (Figure 3A). As shown in Figure 3B, the viral RNA levels were significantly reduced in STAG2^−^^/^^−^ cells compared to WT cells. Additionally, the inhibitory effect of STAG2 knockout on the PDCoV replication was confirmed by the reduced level of PDCoV N protein expression as determined by Western blot analysis (Figure 3C). Importantly, PDCoV infectivity was significantly decreased (~1 log) in STAG2^−^^/^^−^ cells compared to WT cells (Figure 3D), indicating that PDCoV replication was significantly reduced in STAG2^−^^/^^−^ cells. 

### 3.4. STAG2 Is Required for PDCoV Replication

**Figure 4:**
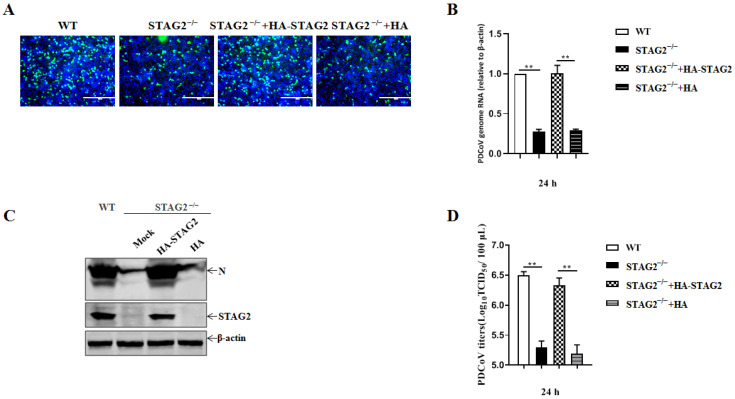
STAG2 is involved in the replication of PDCoV. (**A**) WT, STAG2^−^^/^^−^, and STAG2^−^^/^^−^ IPEC-J2 cells transduced with HA-tagged STAG2 were infected with PDCoV (MOI = 1), the cell monolayer was fixed and stained for PDCoV-N protein (green) and nuclei (blue) for evaluation by IFA; (**B**) WT, STAG2^−^^/^^−^, and STAG2^−^^/^^−^ IPEC-J2 cells transduced with HA-STAG2 were infected with PDCoV (MOI = 1) and viral N mRNA level was measured at 24 h.p.i. by quantitative RT-PCR; (**C**) WT, STAG2^−^^/^^−^, and STAG2^−^^/^^−^ IPEC-J2 cells transduced with HA-STAG2 were infected with PDCoV (MOI = 1) followed by samples detection by Western blot; (**D**) WT, STAG2^−^^/^^−^, and STAG2^−^^/^^−^ IPEC-J2 cells transduced with HA-STAG2 were infected with PDCoV (MOI = 1), virus yield was measured by TCID_50_. The results are representative of three independent experiments (the means ± SD). **, *p* < 0.01. The *p* value was calculated using Student’s *t*-tests.

To further confirm the effect of STAG2 on PDCoV infection, the infectivity of PDCoV was evaluated, followed by exogenous expression of WT STAG2 in STAG2^−^^/^^−^ IPEC-J2 cells. The STAG2^−^^/^^−^ cells were transfected with HA-tagged STAG2 plasmids (HA-STAG2) or an empty vector as a control (vector con) for 24 h, and then, the cells were inoculated with PDCoV and cultured for an additional 24 h. Susceptibility to PDCoV infection was restored upon exogenous expression of WT STAG2 in STAG2^−^^/^^−^ IPEC-J2 cells, suggesting that the effect was specifically due to the loss of STAG2. (Figure 4A). Additionally, an obvious increase in PDCoV N mRNA amount relative to the amount in the vector control was proven by quantitative RT-PCR analysis in STAG2^−^^/^^−^ cells transfected with STAG2 plasmids (Figure 4B). Exogenous expression of WT STAG2 resulted in increase PDCoV replication, as detected by comparing the level of viral nucleocapsid (N) protein in exogenous expression of WT STAG2 in STAG2^−^^/^^−^ IPEC-J2 cells to that in the STAG2^−^^/^^−^ IPEC-J2 cells (Figure 4C). Furthermore, an apparent increase in progeny virus was determined by TCID_50_ assay in the PDCoV-infected STAG2^−^^/^^−^ IPEC-J2 cells transfected with HA-STAG2. Taken together, these data suggest that the loss of STAG2 likely leads to an alteration of signaling pathways within host cells that is commonly shared by PDCoV.

### 3.5. Loss of STAG2 Activates IFN and ISG Expression

**Figure 5:**
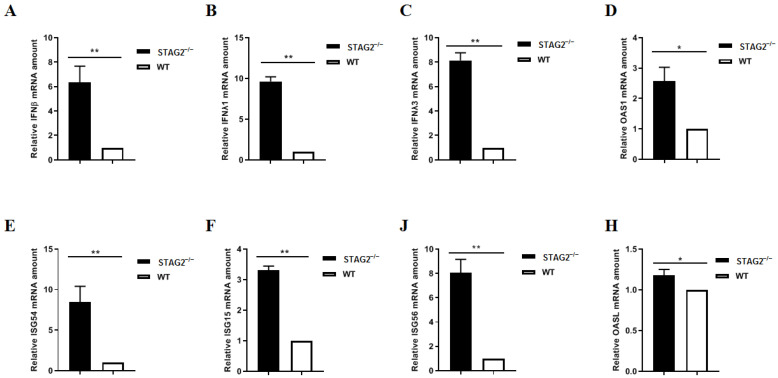
Loss of STAG2 triggers excessive IFN production. (**A**–**H**) The mRNA levels of antiviral genes, IFN-β, IFN-λ1, IFN-λ3, OAS1, IL-54, IL-15, IL-56, and OASL, were examined by quantitative RT-PCR. The results are representative of three independent experiments (the means ± SD). * *p* < 0.05, **, *p* < 0.01. The *p* value was calculated using Student’s *t*-tests.

To identify the mechanism by which the loss of STAG2 leads to a suppression of PDCoV growth, we first performed an unbiased RNA-sequencing analysis, using two different platforms, to profile the transcriptome of WT and STAG2^−^^/^^−^ IPEC-J2 cells. Gene ontology pathway analysis revealed a distinct IFN signature in the STAG2^−^^/^^−^ IPEC-J2 cells (data not shown). Several antiviral proteins in STAG2^−^^/^^−^ IPEC-J2 cells, including IFN-β, IFN-λ1, IFN-λ3, OAS1, IL-54, IL-15, IL-56, and OASL, were significantly increased, as determined by quantitative RT-PCR (Figure 5A–H). 

### 3.6. STAG2 Deletion Triggers IFN Production by Activating the Levels of Phosphorylated STAT1

**Figure 6:**
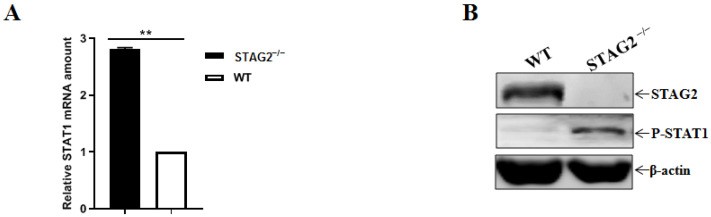
STAG2 deficiency activates the JAK-STAT signaling. (**A**) WT and STAG2^−^^/^^−^ cells were analyzed by quantitative RT-PCR. The results are representative of three independent experiments (the means ± SD). **, *p* < 0.01. The *p* value was calculated using Student’s *t*-tests; (**B**) WT and STAG2^−^^/^^−^ cells were analyzed by Western blot with the indicated antibodies.

We next sought to determine mechanistically how the cell-intrinsic IFN activation occurred in the STAG2^−^^/^^−^ cells. We assayed the phosphorylation status of signaling pathways, based on the IFN-stimulated response elements to induce the expression of IFN-stimulated genes, which establish an antiviral state. The relative quantities of STAT1 mRNA in STAG2^−^^/^^−^ cells relative to the expression in WT cells were up-regulated by quantitative RT-PCR (Figure 6A). Strong phosphorylation of STAT1 was observed in STAG2^−^^/^^−^ cells by Western blot analysis (Figure 6B).

## 4. Discussion

The innate immune system is the first line of the host defense program against pathogens and harmful substances. Antiviral innate immune responses can be triggered by multiple cellular receptors sensing viral components. The activated innate immune system produces IFNs and cytokines that perform antiviral functions to eliminate invading viruses [33,34,35,36]. However, during coevolution with their host, viruses have developed new strategies to evade host antiviral defense programs [37,38,39,40]. 

Coronaviruses has acquired multiple mechanisms to antagonize the host innate immune system by either targeting viral sensors or blocking downstream antiviral signaling molecules. For example, Nsp1 proteins of Severe acute respiratory syndrome coronavirus (SARS-CoV), Middle East respiratory syndrome coronavirus (MERS-CoV), murine hepatitis virus (MHV), TGEV and PEDV suppresses host gene expression [41,42,43]. Of the several known viral evasion strategies, the cleavage of crucial innate immune molecules including adaptors, kinases, and transcriptional factors are considered to be a particularly powerful way for viruses to escape the innate immune response. The 3C-like protease of PEDV and PDCoV, disrupts type I IFN signaling by cleaving the NF-κB essential modulator (NEMO) [44,45]. In addition, PDCoV nsp5 antagonizes type I IFN signaling by cleaving STAT2, an essential factor for IFN responses [46]. IFNs generate an antiviral state through ISG induction as a defense mechanism against viral infection. To combat these antiviral effects of ISGs, many viruses, including CoVs, have evolved elaborate mechanisms, such as altering subcellular localization or inducing ISG degradation, to antagonize their antiviral functions. To our knowledge, some proteins encoded by CoVs, such as PEDV N protein, PEDV nsp1, PDCoV nsp5, PDCoV nsp6, and MHV nsp15, have been demonstrated to hijack IFN signaling to reduce ISG production indirectly [47]. However, viruses are not limited to the aforementioned strategies to antagonize IFN responses.

Cohesin is a multi-subunit nuclear protein complex that coordinates sister chromatid separation during cell division. Highly frequent somatic mutations in genes encoding core cohesin subunits have been reported in multiple cancer types, and its loss of function has been believed to induce aneuploidy [48]. STAG2, a cohesin family gene, is among the most recurrently mutated genes in cancer [49,50]. In contrast to the implication of STAG2 in cancer, less information has been reported on the interplay between STAG2 and microorganism infection. It has been reported that the loss of STAG2, an important component of the cohesin complex, confers resistance to RV replication in cell culture and human intestinal enteroids. In addition, STAG2 deficiency results in spontaneous genomic DNA damage and robust IFN expression via the cGAS-STING cytosolic DNA-sensing pathway. The resultant activation of JAK-STAT signaling and ISG expression broadly protects against virus infections, including RVs [26].

In the present study, we first identified that PDCoV and VSV replication were significantly reduced in the STAG2^−^^/^^−^ cells by establishing of STAG2-knockout IPEC-J2 cell line. To identify the mechanism by which the loss of STAG2 leads to a suppression of PDCoV growth, we first performed an unbiased RNA-sequencing analysis, using two different platforms, to profile the transcriptome of WT and STAG2^−^^/^^−^ IPEC-J2 cells. RNA-seq dataset revealed that STAG2 depletion elicits an excessive IFN expression. Moreover, STAG2 deficiency results in robust IFN expression via the JAK-STAT signaling pathway. Our work may facilitate a better understanding of PDCoV infection and pathogenesis.

## Figures and Tables

**Table 1:** Primers used in this study.

Primer	Forward (5’→3’)	Reverse (5’→3’)
qIFNβ	CCATCTATGAGATGCTCCAG	TCCTTAGGATTTCCACTCTG
qIFNλ1	CCACGTCGAACTTCAGGCTT	ATGTGCAAGTCTCCACTGGT
qIFNλ3	CCAAGGATGCCTTTGAAGAGT	CTGCTGTGCAGGGATGAGTT
qISG15	ATCACCCAGAAGATCGGCG	TCGAAGGTCAGCCAGAACAG
qISG54	CATTGACCCTCTGAGGCAAG	AGCGTGTCCTATTAGTTCC
qISG56	CATACATTTCCACTATGG	TACTCCAGGGCTTCATTCA
qOAS1	CTAGTCAAGCACTGGTACCA	ATCACAGGCCTGGGTTTCGT
qOASL	TCCCTGGGAAGAATGTGCAG	CCCTGGCAAGAGCATAGTGT
qSTAT1	CAGAACGGAGGCGAACCTTA	AGGTTCTGGGGCTTCCTTTG
qPDCoV	AGCAACCACTCGTGTTACTTG	CAACTCTGAAACCTTGAGCTG
qGAPDH	CCTTCCGTGTCCCTACTGCCAAC	GACGCCTGCTTCACCACCTTCT
STAG2-sgRNA	CACCGGTTAATTGTATATACTGTGG	AAACCCACAGTATATACAATTAACC

## Data Availability

Not applicable.
